# Round the Hospitals

**Published:** 1916-04-01

**Authors:** 


### Round the Hospitals.

The Editor thanks the readers of this section, and especially the matrons and sisters, who have this week come forward in increasing numbers with excellent copy. If only the habit is acquired of reading The Hospital and acting after doing so by sending some item of news or experience in the work, or the story of a child's characteristics, or a patient's incident, or a felt want, the combined goodwill and interest of the majority of matrons and sisters throughout the United Kingdom and the Empire should speedily make these pages bright and interesting to everybody. This will ensure them a welcome everywhere every week, when work is done and the period for restful enjoyment, however brief, arrives. The Editor invites his readers to get all the help they can out of him, as he hopes to get reciprocal co-operation and copy out of them. After all, this is the Matrons' and Sisters' section, and our ambition is to make it in fact not only helpful, but just what they would wish it to be.

---

There was great joy at the Hampstead Military Hospital when an invitation arrived from Buckingham Palace for fifty of the brave men there to be guests of their Majesties on March 23. Needless to say, the morning was spent in preparation for the very special "party," and very smart they looked as they drove away with the matron and a sister to share the honours. A few Colonials were amongst the guests, who were specially cheered by really seeing and talking to the King for whom they have fought so bravely. The arrangements consisted of tea—a good sit-down tea such as the soldier loves—in the course of which their Majesties and other members of the Royal Family welcomed their guests, and never tired of shaking hands and writing their much-appreciated autographs on the programmes. Tea was followed by a variety performance and some good old songs, after which each section made for home. Certainly a happier day was never spent, and it is one which will be always gratefully remembered by the "Hampstead warriors."

On the 21st ult. Sir Frederick Milner visited the base hospital at Whitworth Street, Manchester, and a number of the branch hospitals, at each of which he addressed the patients, telling them how keenly the King and Queen appreciate the splendid service they have rendered and how anxious their Majesties are that all that is possible should be done to alleviate their sufferings and to expedite their recovery. He explained to the men the work of the Soldiers' and Sailors' Help Society, an organisation which has for its object the giving of financial assistance to disabled soldiers during the time that elapses between their discharge and the first payment of their pension. Before Sir Frederick left the base hospital he was asked by one of the patients there to tell their Majesties that the wounded soldiers had been more than compensated for what they had gone through by the kindness they had received from British nurses since their return from the Front.

---

On one of the surprise visits to hospitals that King George V. habitually makes, the Royal party was greeted at the door of a ward by the sister. Looking at him in a puzzled way, she went up to His Majesty and asked if he would like to be shown round the ward. "Your face, Sir," she said, "seems familiar, but I can't put a name to it." The King smiled a little and replied by asking questions about the patients. It was not until His Majesty had departed that the sister realised to whom she had been talking.

***** It is our invariable practice to pay for all items of news which we may publish. The minimum payment is 5s. Paragraphs of about twenty lines in length—that is to say, of 150 words or less—are preferred. In this section during the war we propose to give 10s. to the correspondent who may send us in any week the best incident connected with hospital life and work. In this way we hope to afford to all practical aid and encouragement; while those who reciprocate will be able to render us invaluable service. Contributions should be addressed to The Editor, The Hospital, 28 and 29 Southampton Street, Strand, London, W.C., and, to be in time for the current week's issue, should reach him by the first post on Mondays.**

